# Enhancing endothelial colony-forming cells for treating diabetic vascular complications: challenges and clinical prospects

**DOI:** 10.3389/fendo.2024.1396794

**Published:** 2024-07-15

**Authors:** Yaqiong Liu, Caomhán J. Lyons, Christine Ayu, Timothy O’Brien

**Affiliations:** Regenerative Medicine Institute (REMEDI), Biomedical Sciences Building, University of Galway, Galway, Ireland

**Keywords:** endothelial colony forming cells, diabetes mellitus, pharmacological conditioning, genetic modification, disease-related cellular dysfunction, clinical translation

## Abstract

Diabetes mellitus (DM) is a metabolic disease characterized by hyperglycemia, leading to various vascular complications. Accumulating evidence indicates that endothelial colony-forming cells (ECFCs) have attractive prospects for repairing and restoring blood vessels. Thus, ECFCs may be a novel therapeutic option for diabetic patients with vascular complications who require revascularization therapy. However, it has been reported that the function of ECFCs is impaired in DM, which poses challenges for the autologous transplantation of ECFCs. In this review, we summarize the molecular mechanisms that may be responsible for ECFC dysfunction and discuss potential strategies for improving the therapeutic efficacy of ECFCs derived from patients with DM. Finally, we discuss barriers to the use of ECFCs in human studies in light of the fact that there are no published reports using these cells in humans.

## 1 Introduction

**Figure 1:**
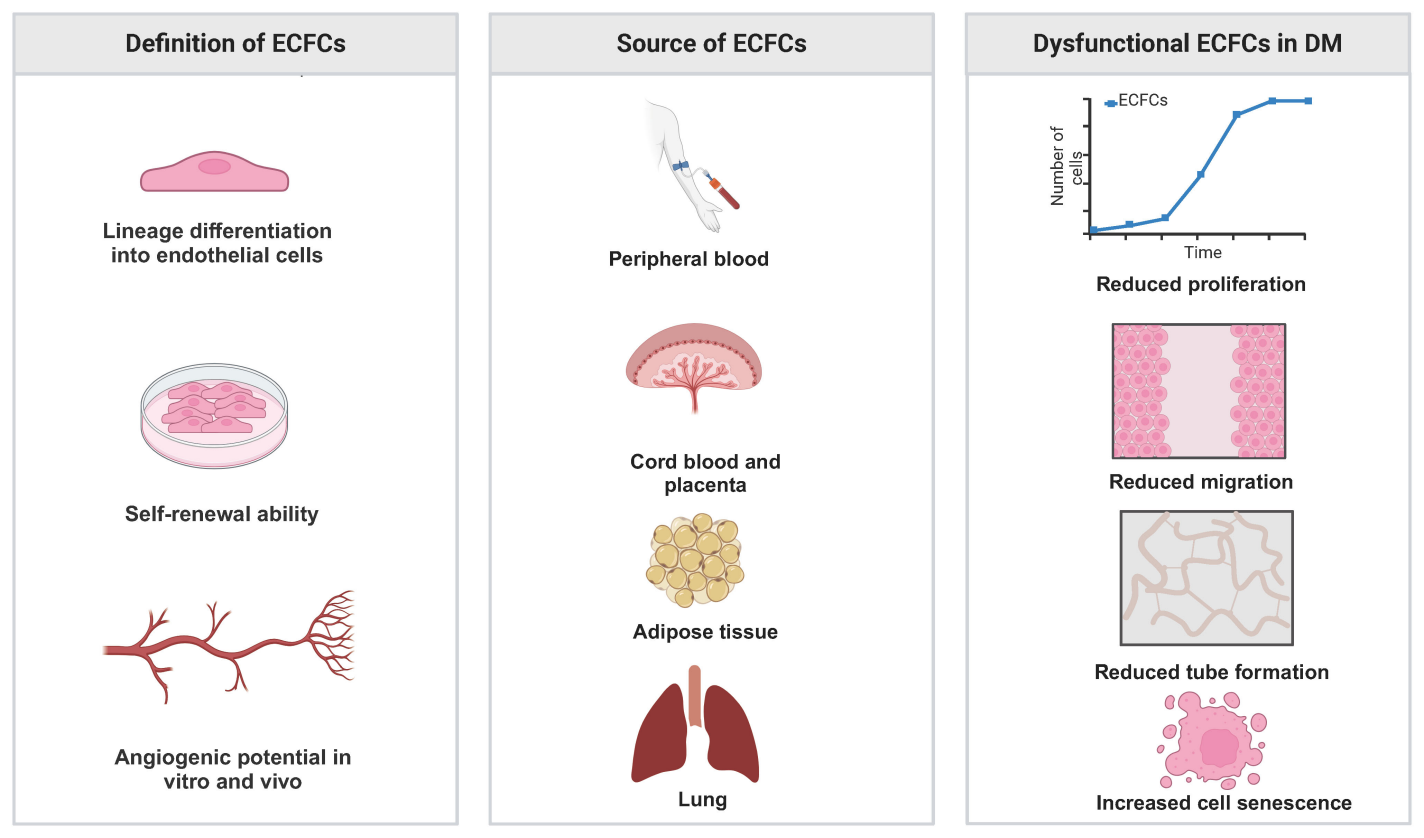
Definition, source of ECFCs and their phenotype in DM.

Diabetes mellitus (DM) is a metabolic disease characterized by long-term hyperglycemia, which can be mainly classified into two subtypes: type 1 DM (T1DM) and type 2 DM (T2DM). T1DM is attributed to the immune-mediated destruction of pancreatic β-cells (1) and T2DM is caused by tissue resistance to insulin and a relative lack of insulin (2). It is estimated that approximately 537 million people are affected by DM worldwide in 2021, with the number expected to rise to 783 million by 2045 (3). DM can lead to macrovascular and microvascular complications, which imposes a significant health burden (4). The macrovascular complications of DM include coronary artery disease, peripheral arterial disease, and stroke. The primary microvascular complications encompass diabetic nephropathy, neuropathy, and retinopathy (5). For patients with severe vascular complications that need revascularization therapy, endothelial colony-forming cells (ECFCs) have emerged as a promising therapy due to their strong angiogenic ability. ECFCs are progenitors of endothelial cells, representing a cell population that has self-renewal ability and the capacity to form functional blood vessels (6). However, previous studies suggest that ECFCs isolated from diabetic patients exhibited functional defects, thereby limiting their therapeutic potential (**Figure 1**) (7, 8). Due to the difficulty in isolating ECFCs from peripheral blood (PB), most studies on ECFCs in diabetic patients have revolved around exploring the biology of ECFCs from gestational diabetes mellitus (GDM) patients. CB-ECFCs from GDM patients exhibit increased senescence, reduced proliferation, migration, and tube formation when compared with those from healthy donors (9–13). Besides, it has been reported that it was more difficult to isolate ECFCs from the PB of T2DM patients than from healthy donors (14). An altered function of PB-ECFCs was also found in diabetic patients with vascular complications. Tan et al. investigated the cell number and function of CD34^+^ CD45^−^ ECFCs between healthy donors and patients with proliferative diabetic retinopathy (PDR) using a cross-sectional cohort design. Their results revealed that PB-ECFCs from patients with PDR had impaired capacity to migrate and were not able to form functional vascular tubes (15). Another study reported that PB-ECFCs isolated from patients with DM with neuroischemic (NI) or neuropathic (NP) foot ulcers exhibited reduced colony formation, proliferation, migration capability, and nitric oxide (NO) bioavailability (16). Hence, it is reasonable to suggest that optimizing the potency of ECFCs in DM by reversal of dysfunction may achieve the desired therapeutic potential. Several strategies have been developed to enhance the efficacy of ECFCs in DM, such as pretreatment with bioactive agents or chemical factors, genetic modification, and combination with other cell types such as mesenchymal stromal cells (MSCs). In this review, we aim to summarize the mechanisms that account for the reduced cell number and compromised function of ECFCs from DM and explore potential strategies to improve the therapeutic efficacy of ECFCs from patients with DM. Due to the dearth of clinical trials using these cells, we also discuss translational barriers.

## 2 Definition and characteristics of human ECFCs

**Figure 2:**
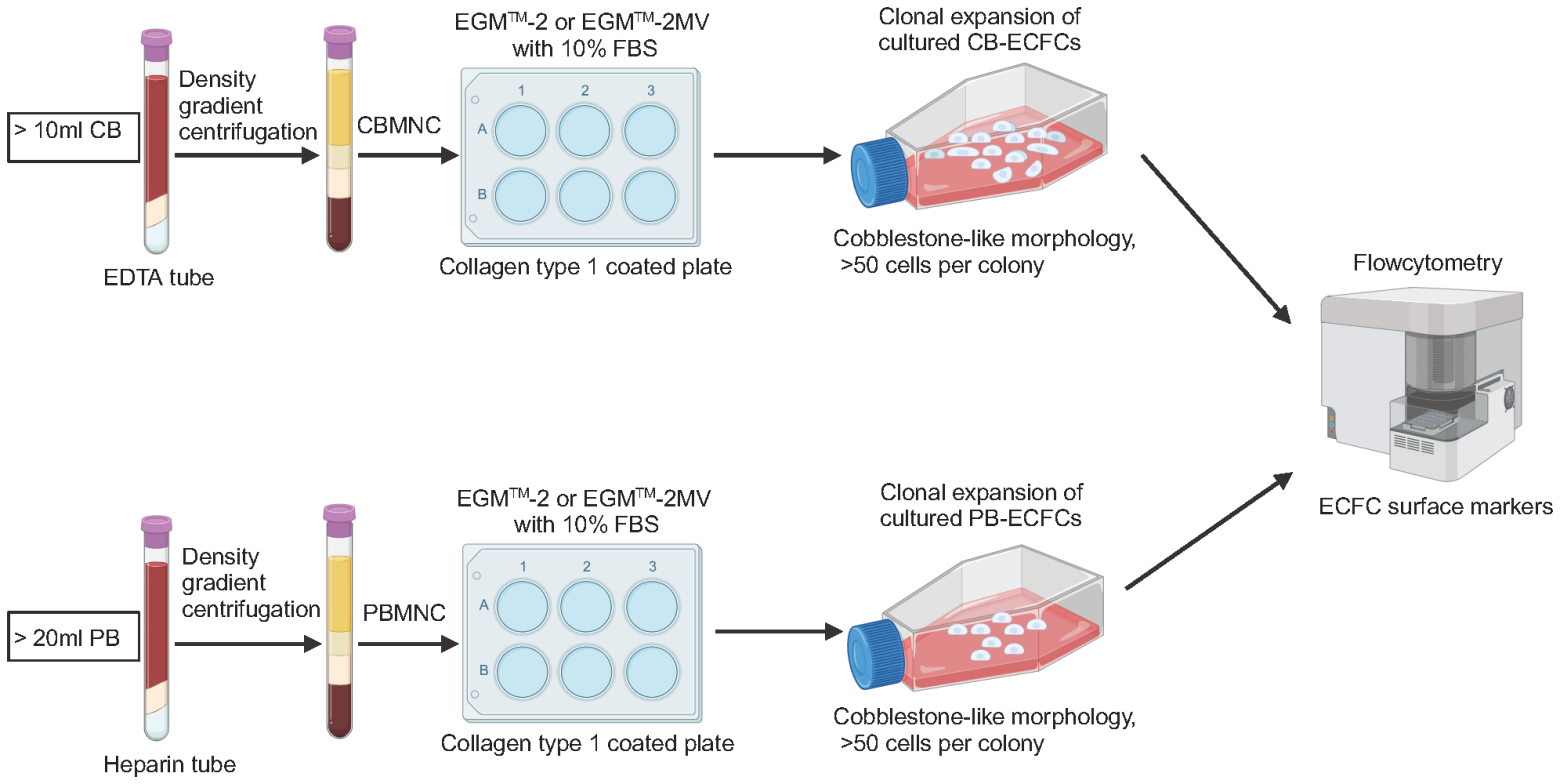
Schematic of isolation, culture, and characterization of ECFCs. CB, cord blood; EDTA, ethylene diamine tetra acetic acid; CBMNC, cord blood mononuclear cell; PB, peripheral blood; PBMNC, peripheral blood mononuclear cell.

In 1997, Asahara et al. first isolated putative endothelial progenitor cells (EPCs) from peripheral blood mononuclear cells (PBMNCs). They reported that EPCs were able to integrate into regenerating host blood vessels within injured areas and contribute to vascular repair (17). Subsequent studies found that these EPCs promoted angiogenesis through a paracrine signaling mechanism, but they had limited proliferative ability and could not form blood vessels directly (18, 19). In 2004, Ingram et al. identified a new cell type termed ECFCs that exhibited superior clonal and angiogenic ability (20). In humans, ECFCs are defined using the following three features: (i) lineage differentiation into endothelial cells; (ii) self-renewal ability; and (iii) angiogenic potential *in vitro* and *in vivo* (21) (**Figure 1**). The first feature emphasizes that ECFCs exhibit the endothelial phenotype after *in vitro* culture, which is used to distinguish ECFCs from other blood outgrown cell types of hematopoietic lineages, such as myeloid angiogenic cells (MACs) (22). The second feature emphasizes the strong self-renewal potential of ECFCs. In some cases, a single ECFC is capable of expanding to a colony with >10,000 cells after 14 days in culture (20, 23). The third feature, angiogenic potential *in vitro* and vivo, is also essential for distinguishing ECFCs from MACs. MACs cannot form blood vessels themselves, but they support vascular repair by secreting angiogenic factors (22). To date, the origin of ECFCs is not well understood. Lin et al. have provided evidence for a bone marrow origin of ECFCs based on the analysis of blood samples from bone marrow transplant recipients who had received gender-mismatched transplants (24). Fujisawa et al. investigated the ECFCs isolated from PB of male patients after a sex mismatched allogeneic bone marrow transplant. They found that these ECFCs exhibited a XY phenotype instead of a XX phenotype, suggesting that ECFCs originated from blood vessels (25). Surprisingly, the two similar studies have reached very different conclusions. However, a recent study suggested that ECFCs originated from blood vessel walls through lineage tracing and single cell RNA sequence analysis (26). Although the origin of ECFCs is still controversial, ECFCs have been successfully isolated from various tissues (**Figure 1**). ECFCs are most frequently isolated from cord blood (CB) and peripheral blood (PB) (27) but can also be derived from other tissues (28–30) (**Figure 1**). CB-ECFCs and PB-ECFCs show differences in gene expression and pro-angiogenic capacity (31, 32). However, the frequency of ECFC is very low in CB and PB: approximately 50 CB-ECFCs per 1x10^8^ cord blood mononuclear cells (CBMNCs) (33) and 1.7 PB-ECFCs per 1x10^8^ PBMNCs (34). It is also important to point out that there is heterogeneity within ECFCs population. Based on the different proliferative potential, CB-ECFCs exhibit a hierarchy of three levels: (i) high proliferative potential ECFC (HPP-ECFC); (ii) low proliferative potential ECFC (LPP-ECFC); (iii) endothelial cell cluster (ECC). HPP-ECFC has the highest proliferative potential and forms colonies containing more than 2000 cells, which gives rise to LPP-ECFC and ECC. LPP-ECFC forms colonies with 50–2,000 cells and ECC forms colonies that contain less than 50 cells (20, 35). In 2019, the International Society on Thrombosis and Hemostasis Congress proposed a standardized protocol for the isolation and expansion of PB-ECFCs and CB-ECFCs without focusing particularly on their surface markers (36) (**Figure 2**). Generally, ECFCs are positive for CD34 (37), CD31, VEcadherin, von Willebrand factor (vWF), CD146, and VEGFR2 expression, whereas they are negative for hematopoietic markers such as CD14 and CD45 (6).

## 3 Mechanisms leading to ECFCs dysfunction in DM

**Figure 3:**
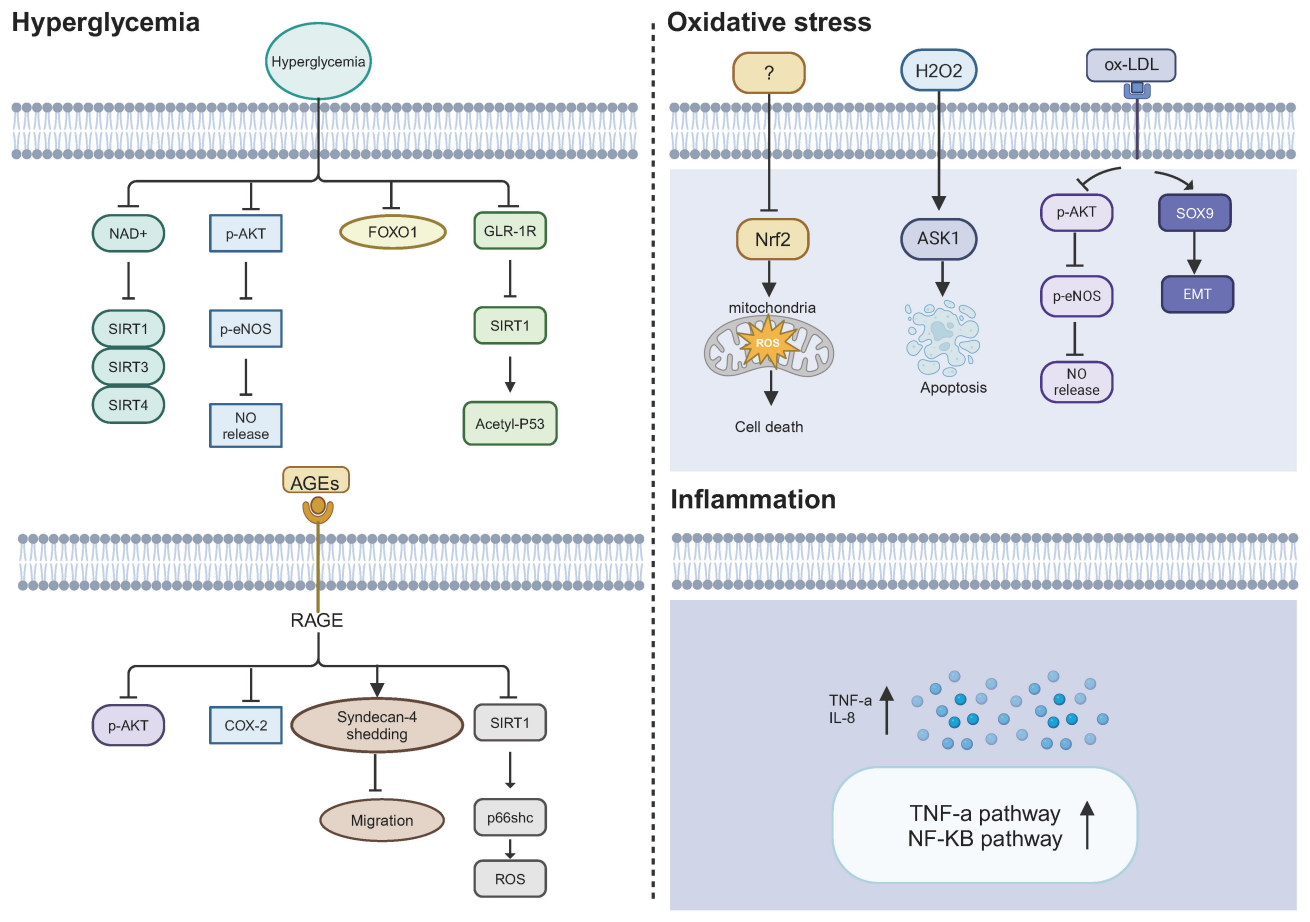
Potential mechanisms causing ECFC dysfunction in DM. Hyperglycemia, oxidative stress, and inflammation may lead to a defective phenotype of ECFCs in a diabetic state. NAD^+^, nicotinamide adenine dinucleotide; SIRT1/3/4, sirtuin 1; 3; and 4; p-AKT, phosphorylated protein kinase B; p-eNOS, phosphorylated endothelial nitric oxide synthase; NO, nitric oxide; FOXO1, fork head box O 1; GLP-1R, glucagon-like peptide 1 receptor; Acetyl-p53, acetylated p53; AGEs, advanced glycation end products; RAGE, receptor for AGEs; COX-2, cyclooxygenase-2; ROS, reactive oxygen species; Nrf2, nuclear factor erythroid 2-related factor 2; ASK1, apoptosis signal-regulating kinase 1; SOX9, SRY-box transcription factor 9; EMT, endothelial-to-mesenchymal transition; IL-8, Interluekin-8.

The triggering factors that lead to dysfunctional ECFCs in DM are still unclear, but hyperglycemia, oxidative stress, and inflammation have been hypothesized as key factors in ECFC dysfunction (**Figure 3**). These three triggering factors act independently and also interact with each other in a vicious cycle, which contributes to the altered function of ECFCs in DM.

### 3.1 Hyperglycemia

Hyperglycemia, a hallmark of diabetes mellitus, may be one of the leading factors that result in ECFC dysfunction in DM. It has been reported that high glucose impaired the function of ECFCs, including colony formation, self-renewal capacity, and tube formation (38). The adverse effects of high glucose on ECFC have been suggested through affecting sirtuin (SIRT) family and nitric oxide (NO) bioavailability. The SIRT family are nicotinamide adenine dinucleotide (NAD)^+^-dependent histone deacetylases, which includes seven members (SIRT1–SIRT7) (39). SIRT1 is the most widely studied member of this family and shows anti-inflammatory and anti-oxidative stress effects and improves the mitochondrial function under stress (40). SIRT1 has been found to protect endothelial cells from stress-induced senescence and improve endothelium-dependent vasodilation by deacetylating endothelial nitric oxide synthase (eNOS) and increasing NO bioavailability (41). As for NO bioavailability, its decreased level is a key factor for endothelial cells dysfunction (42). There are several possible mechanisms for decreased NO bioavailability, including reduced eNOS mRNA or protein expression, endogenous competitive inhibitors of L-arginine, reduced tetrahydrobiopterin (BH4) level, and interaction between NO and superoxide (43). Due to the important role of SIRT1and NO bioavailability in endothelial cells, researchers have also investigated their alteration in ECFCs exposed to high glucose. Chen et al. demonstrated that high glucose (25 mM glucose) resulted in increased senescence and impaired proliferation, migration and tube formation ability of PB-ECFCs. Mechanistically, high glucose suppressed phosphorylation of Akt and eNOS, thereby reducing NO bioavailability. Besides, high glucose inhibited the activity of FOXO1 in PB-ECFCs. The negative effects of high glucose could be reversed by co-incubation with the NO donor sodium nitroprusside or p38 mitogen–activated protein kinase (MAPK) inhibitor. Conversely, the negative effects of high glucose could be enhanced by co-incubation with NOS inhibitor l-Ng-nitro-l-arginine methyl ester (l-NAME) or PI3K inhibitor LY294002. These results suggested that high glucose may impair ECFC function by modulating PI3K/Akt, NO, and p38 MAPK-related mechanisms (44). Likewise, Huang et al. reported that high glucose levels resulted in cellular senescence, defective migration, and tube formation of PB-ECFCs, as well as decreased phosphorylation of Akt and eNOS (45). Glucagon-like peptide-1 (GLP-1) plays a key role in regulating blood glucose homeostasis through GLP-1 receptor (GLP-1R) (46). Importantly, the activation of GLP-1R was able to increase NO production in endothelial cells and blunted high glucose-induced endothelial dysfunction (47). Therefore, Tu et al. investigated whether GLP-1R and SIRT1 led to PB-ECFCs dysfunction under high glucose conditions. They found that GLP-1R and SIRT1 were downregulated in PB-ECFCs under high glucose conditions (25 mM glucose). The knockdown of GLP-1R and SIRT1 aggravated the dysfunction of PB-ECFCs, including increased apoptosis, and impaired migration, adhesion and angiogenic abilities. Moreover, the upregulation of GLP-1R improved the dysfunctional ECFCs by regulating SIRT1 expression (48). Consistent with the aforementioned study, another group showed decreased transcription levels of *SIRT1*, *SIRT3*, and *SIRT4* in CB-ECFCs after high glucose treatment (30 mM glucose) (49).

Persistent hyperglycemia increases the formation of advanced glycation end-products (AGEs) (50). AGEs are the products of non-enzymatic glycation of macromolecules (proteins, lipids, and nucleic acids) with monosaccharides such as glucose, glyceraldehyde, and fructose (51). AGEs are associated with increased oxidative stress (52) and inflammation (53). Chen et al. revealed that AGEs promoted the apoptosis of ECFCs and reduced the migration and tube formation of ECFCs. They investigated the effects of AGEs on Akt/eNOS and cyclooxygenase-2 (COX-2) protein expression as these proteins play an important role in endothelial cell homeostasis. The results showed that AGEs increased the expression of the receptor for AGE (RAGE) and decreased Akt and COX-2 protein expression. However, RAGE did not influence the mRNA nor protein levels of eNOS. These results provided evidence that Akt and COX-2 expression may be involved in the mechanism underlying this impairment induced by RAGE (54). Another study showed that AGEs accelerated the shedding of syndecan-4 (synd4, a ubiquitous heparan sulfate proteoglycan receptor), thereby impairing the migration of ECFCs. Besides, the lack of synd4 led to poor homing of ECFCs to the site of injury in lower limb ischemic mice (55). Additionally, a study reported that AGEs were able to promote oxidative stress via SIRT1/p66^shc^ pathway and stimulate the upregulation of high mobility group box-1 protein (HMGB-1). The inhibition of HMGB-1 (a pro-inflammatory cytokine) attenuated oxidative stress induced by AGEs. Conversely, the effects of oxidative stress could be exacerbated by a positive feedback of which can bind to RAGEs, facilitating more ROS production (56).

Overall, mechanisms implicated in hyperglycemia-induced ECFCs dysfunction include: PI3K/Akt/eNOS pathway, the decreased SIRT1 level, and AGEs-RAGE axis. Previous studies have demonstrated that hyperglycemia causes endothelial dysfunction by four major mechanisms: (i) increased glucose flux into the polyol pathway; (ii) increased intracellular production of advanced glycation end-products (AGEs); (iii) activation of protein kinase C (PKC) isoforms; (iv) overactivity of the hexosamine pathway [see review (57)]. Therefore, it is worth exploring whether hyperglycemia could activate the polyol, hexosamine pathway, and PKC isoforms to cause ECFC damage (58).

### 3.2 Oxidative stress

Oxidative stress is caused by the imbalance between ROS production and ROS scavenging, which negatively affects the survival of cells (59). The important sources of ROS production include mitochondria, NADPH oxidases, xanthine oxidases, cyclooxygenases, and uncoupled endothelial (60). ROS are highly reactive oxidizing agents characterized by the presence of one or more unpaired electrons, such as superoxide anion (O2•−), hydroxyl radical (•OH), and H_2_O_2_ (61). ROS at low concentrations can serve as signaling molecules which transmit signals for normal physiological processes, such as cell growth and cellular adaptation responses (62). Excessive ROS would reduce NO bioavailability, activate inflammatory pathways mediated by TNF-α and NF-κB, leading to endothelial cell dysfunction (42). Notably, it has been reported that ROS was increased in ECFCs from patients with DM (63), which may play an important role in diabetes-induced ECFC dysfunction. But there is little evidence on how increased ROS levels disturb ECFC function in DM (64). A study found that increased ROS level in DM-ECFCs was mainly attributed to mitochondrial dysfunction. DM-ECFCs exhibited mitochondrial fragmentation and dysfunction accompanied by downregulation of nuclear factor erythroid 2-related factor 2 (*Nrf2*). *Nrf2* is a key regulator of the cellular antioxidant system and the overexpression of *Nrf2* improved the mitochondrial fragmentation and dysfunction by influencing protein associated with mitochondrial fission and fusion in DM-ECFCs (63). With the exception of decreased Nrf2 levels, other molecular mechanisms are involved in oxidative stress-mediated ECFCs dysfunction. Using the oxidative stress model induced by hydrogen peroxide (H_2_O_2_), Ingram et al. demonstrated that oxidative stress promoted apoptosis of ECFCs and diminished their tube formation *in vitro* and *in vivo* by activating apoptosis signal-regulating kinase 1 (ASK1) (35). ASK1 was shown previously to be negatively regulated by redox-sensitive binding partners (65). Furthermore, H_2_O_2_ treatment impaired the vessel formation of ECFCs *in vivo* (35). Another study showed that oxidative levels of five proteins (T-complex protein 1 subunit α, isoform A of prelamin-A/C, cofilin-1, peroxiredoxin-4, and actin) were upregulated in H_2_O_2_ induced ECFCs, which provided novel insight into the proteomic mechanisms of oxidative stress on ECFCs (66). Additionally, the generation of oxidized low-density lipoprotein (oxLDL) would increase under conditions of excessive oxidative stress (67). OxLDL has been shown to impair the growth and bioactivity of ECFCs, decrease Akt phosphorylation and eNOS protein expression in a dose-dependent manner, and increasing lectin-like OxLDL receptor protein expression (68). A very recent study found that ECFCs underwent endothelial-to-mesenchymal transition (EndoMT) and exhibited a loss in self-renewal and proliferative capacity after OxLDL stimulation (69). Collectively, oxidative stress impaired ECFC function by a variety of mechanisms, including causing mitochondrial fragmentation and dysfunction, increasing apoptosis, decreasing Akt phosphorylation and eNOS protein expression, and increased EndoMT. While oxidative stress has been recognized as a primary cause in endothelial cell dysfunction, its importance in ECFC dysfunction induced by diabetes requires further confirmation.

### 3.3 Inflammation

DM is associated with an inflammatory state, caused by complex factors, including increased levels of ROS, oxidized lipids, increased angiotensin II, free fatty acids, AGEs and reduced NO level (70). These factors lead to increased levels of plasma inflammatory cytokines in DM, including C-reactive protein (CRP) (71), interleukin-6 (IL-6), interleukin 1β (IL-1β), interleukin 8 (IL-8) (72), tumor necrosis factor-α (TNF-α), and so on (73). Theses inflammatory cytokines bind to various receptors that trigger a common pathway of mediators, like oxidative stress and NF-κB pathway, in endothelial cells, which eventually result in endothelial cell dysfunction (74). This paragraph aims to discuss the impact of inflammatory cytokines on ECFCs. Studies suggested that the inflammatory cytokines can lead to dual effects in ECFCs: (i)low level of inflammatory cytokines stimulated the proliferation of ECFCs (75); (ii) high level of inflammatory cytokines impaired the function of ECFCs (76). Two studies have reported that high concentrations of TNF-α (>10ng/ml) significantly inhibited the proliferation and tube formation of ECFCs and increased the apoptosis of CB-ECFCs (76, 77). But the mechanisms underlying the TNF-α-induced impairment of CB-ECFCs remains unclear. The interaction between TNF-α and transmembrane receptor 1 may be involved in the impairment of CB-ECFCs (78). Shen et al. provided additional evidence for inflammation induced ECFC dysfunction in DM. They compared the differentially expressed genes (DEGs) between healthy PB-ECFCs and diabetic PB-ECFCs using microarray data. Pathway analysis revealed that these DEGs were mainly associated with inflammatory pathways, such as “NF-κB signaling pathway” and “TNF-α signaling pathway”. Additionally, IL-8 (a pro-inflammatory chemokine) was upregulated in DM-ECFCs compared with healthy-ECFCs (79). However, another study has reported that increased plasma inflammatory cytokines in diabetic patients with chronic kidney disease did not influence the success rate of PB-ECFC isolation (80). Nevertheless, this study did not rule out the influence of inflammatory cytokines on other aspects of the biology of ECFCs.

Overall, inflammation could be caused by multiple factors, which leads to the increased inflammatory cytokines level in DM. These inflammatory cytokines could alter gene expression patterns of ECFCs and activate inflammatory pathways. These changes ultimately result in decreased tube formation and increased apoptosis of ECFCs.

## 4 Strategies for improving the therapeutic efficacy of ECFCs in DM

**Figure 4:**
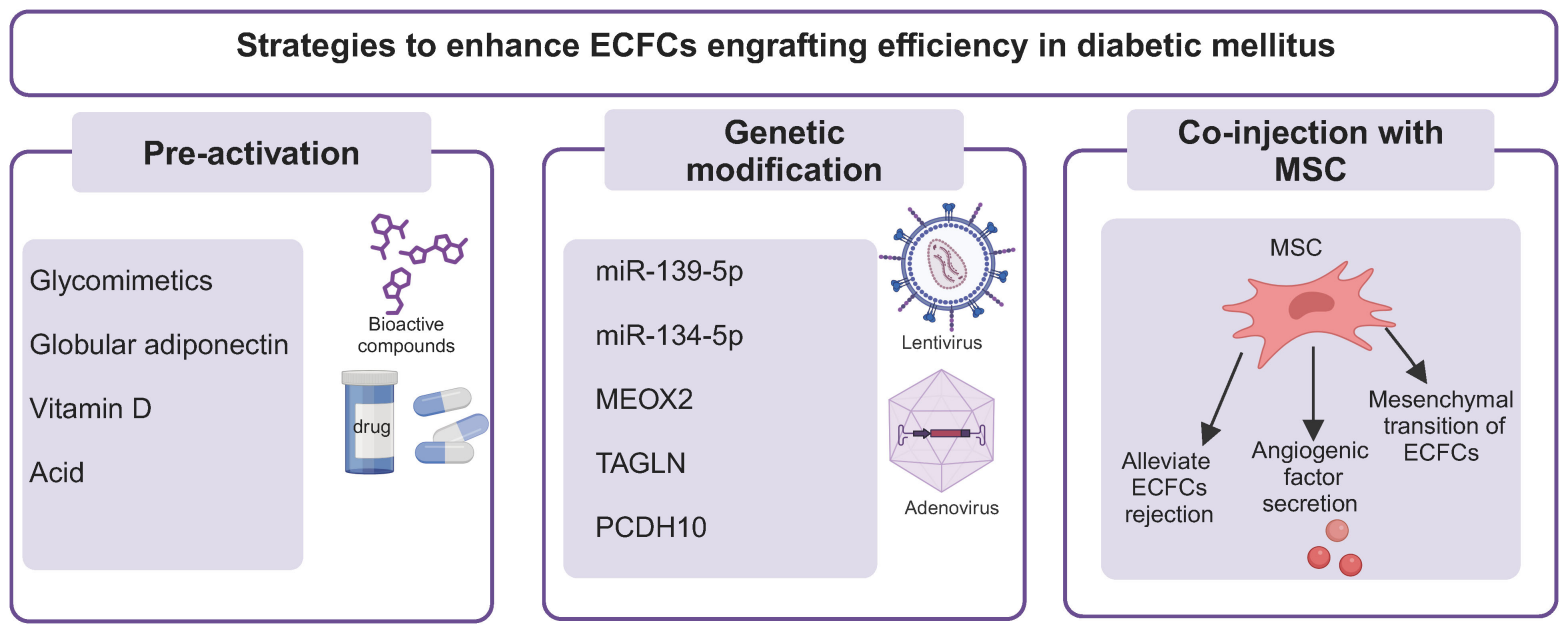
Strategies to enhance the therapeutic efficacy of ECFCs in DM. Several strategies have been used to improve the therapeutic efficiency of ECFCs in DM, including pretreatment of ECFCs with bioactive compounds or chemical factors (pH), genetic modification, and co-injection with MSCs. ECFCs, endothelial colony forming cells; *MEOX2*, mesenchyme homeobox 2; *TAGLN*, transgelin; *PCDH10*, protocadherin 10; MSC, mesenchymal stem cells; VEGFA, vascular endothelial growth factor A; FGF2, fibroblast growth factor-2.

To achieve the desired therapeutic potential, several strategies have been proposed to improve the compromised function of ECFCs in DM. These strategies can be mainly divided into three categories: (i) pretreatment of ECFCs with biological compounds; (ii) genetic modification; (iii) co-injection with mesenchymal stem cells (MSCs) (**Figure 4** and **Table 1**).

**Table 1:** Strategies for improving the therapeutic efficacy of ECFCs in DM.

Strategies		Assessment methods for angiogenesis	Main results	References
**Pretreatment with biological compounds**	Acidic conditions	*In vitro* Matrigel assay; Laser Doppler blood flow analyzer in a hind limb ischemia (HLI) mouse model; Histological analysis	Acidic preconditioning improved the survival, migration and tube formation ability of ECFCs under high glucose condition; Preconditioned ECFC had superior ability to restore hind limb revascularization and reduce ischemia-related tissue damage and inflammation in mouse models of T2DM	(89)
	Globular adiponectin	Laser Doppler blood flow analyzer in a HLI mouse model Intravital near-infrared fluorescence imaging	Pretreating ECFCs with globular adiponectin improved ECFC function *in vitro* and *in vivo* in normoglycemic and hyperglycemic environments	(85)
	Glycomimetic C3	*In vitro* Matrigel assay Wound healing assay	Glycomimetic C3 enhanced the migration and tube formation of PB-ECFCs from diabetic patients with NI	(16)
	Vitamin D	*In vitro* Matrigel assay	Vitamin D alleviated the dysfunction of ECFCs from pregnancies with GDM or healthy ECFCs exposed to hyperglycemia.	(10)
**miRNA Modification**	miR-134–5p	*In vitro* Matrigel assay; Laser Doppler Perfusion Imager system	miR-134–5p was upregulated in diabetic PB-ECFCs; Knockdown of miR-134–5p could restore the impaired migration and angiogenic activities of diabetic PB-ECFCs via upregulation of NRIP1; FIR pretreatment promoted the angiogenic ability of dysfunctional PB-ECFCs induced by high glucose in a mouse ischemic limb model; The protective effects of FIR could be reduced by the overexpression of miR-134–5p	(93)
	miR-139–5p	*In vitro* Matrigel assay; Matrigel plug assay *in vivo*; PeriCam Perfusion Speckle Imager; Histological analysis; Count the number of CM-DiI-labeled PB-ECFCs in the ischemic muscle	miR-139–5p was upregulated in PB-ECFCs from diabetic patients and in healthy PB-ECFCs exposed to high glucose; miR-139–5p inhibited VEGF and PDGF-B expressions by c-jun to impair the ECFC function; miR-139–5p prevented tube formation *in vivo*; Knockdown of miR-139–5p promoted ECFC-mediated angiogenesis and blood perfusion in hind limb ischemia in diabetic mice	(92)
**mRNA Modification**	*MEOX2*	Matrigel assay	Compared with healthy pregnancies, *MEOX2* was upregulated in pregnancies complicated by T1DM and T2DM; Knockdown of *MEOX2* in CB-ECFCs from DM pregnancies led to decreased network formation	(94)
	*PCDH10*	Matrigel assay	Increased *PCDH10* expression was observed in GDM-ECFCs; Knockdown of *PCDH10* recovered the impaired proliferation, migration, adhesion, and tube formation ability of GDM-ECFCs	(12)
	*TAGLN*		Upregulation of *TAGLN* was found in GDM-ECFCs; Knockdown of *TAGLN* expression improved migration and network formation in GDM- ECFCs	(11)
**Co-Injection with MSCs**	MSCs	*In vitro* Matrigel assay; Matrigel plug assay *in vivo*	MSCs enhanced the vasculogenic capacity of ECFCs to form functional microvessels under *in vitro* and *in vivo* hyperglycemic conditions	(104)

### 4.1 Pretreatment of ECFCs with biological compounds

#### 4.1.1 Pretreatment of ECFCs with glycomimetics

A previous study has demonstrated the defects in ECFCs from patients with diabetes with NI or NP ulcers (16). In an ex vivo study, it was suggested that glycomimetic C3 was able to enhance the migration ability of ECFCs from diabetic patients with NI and NP. Nevertheless, glycomimetic C3 only enhanced the tube formation in ECFCs from diabetic patients with NI. Although the mechanism by which glycomimetic C3 improved the function of ECFCs in diabetic patients was unclear, a previous study has shown that glycomimetic C3 exerted a protective effect in an endothelial model of lipid-induced oxidative stress through the Nrf2/ARE and Akt/eNOS signaling pathways (81).

#### 4.1.2 Pretreatment of ECFCs with globular adiponectin

The literature has shown that individuals with higher serum adiponectin levels are less likely to develop insulin resistance and T2DM (82, 83). In addition, adiponectin exerts vasculoprotective actions by increasing NO production and inhibiting endothelial apoptosis (84). Leicht et al. investigated the effects of gAd (an active domain of adiponectin) on diabetic ECFCs and found that pre-treatment of diabetic ECFCs with gAd increased neovascularization in a diabetic hindlimb ischemia mouse model. Thus, preconditioning of DM-ECFCs with gAd may be a novel approach to counter their dysfunction in DM (85).

#### 4.1.3 Pretreatment of ECFCs with vitamin D

Emerging evidence has suggested that vitamin D deficiency is associated with endothelial dysfunction (75). Vitamin D supplementation has antioxidant capacity and anti-inflammatory effects to rescue dysfunctional endothelial cells (76). Gui et al. showed that pre-activation of ECFCs with vitamin D improved the migration and tube formation of ECFCs from pregnancies with GDM (10). However, this study did not investigate the effects of vitamin D on diabetic ECFCs *in vivo*. Additionally, the effectiveness of vitamin D with a longer duration of supplementation and at different doses needs to be studied in the future to fully explore the potential of this therapy.

#### 4.1.4 Pretreatment of ECFCs in acidic conditions

Reduced blood flow during tissue injury tends to drive cell metabolism into anaerobic glycolysis, which leads to lactate formation and pH reduction. Acidosis reduces cell proliferation and viability (86). Acidic preconditioning has been found to be a potential strategy to improve the cell viability in the host acidic environment (76). Mena et al. demonstrated that acid preconditioning enhanced the proliferation and tube formation of ECFCs under pro-inflammatory and high glucose condition. Compared with PBS treatment and non-preconditioned ECFCs, the transplantation of acidic preconditioned ECFCs increased the capillary density and diminished the inflammation score in the murine model of the diabetic ischemic limb (87). However, more experiments are needed to explore the different PH levels of cell culture and incubation time (88, 89) to obtain more beneficial effects and regenerative capacity of ECFCs.

### 4.2 Genetic modification

#### 4.2.1 miRNAs

miRNAs are endogenous non-coding RNAs of 21–25 nucleotides that primarily affect the post-transcriptional regulation of target genes by binding to the 3’ untranslated region of mRNA (90). Luo et al. found that miR-139–5p, an anti-angiogenesis miRNA (91), was upregulated in diabetic ECFCs compared to ECFCs from healthy control patients. *In vitro* experiments showed that the inhibition of miR-139–5p can rescue the impaired migration and tube formation ability of diabetic ECFCs. Matrigel plug assays demonstrated that the inhibition of miR-139–5p facilitates restoration of the blood vessel-forming ability of diabetic ECFCs *in vivo*. Mechanistically, miR-139–5p was found to target the transcription factor *c-jun*, thereby decreasing the expression of VEGF/PDGF-B (92). Another study reported that miR-134–5p was more highly expressed in diabetic ECFCs compared to ECFCs from disease free controls, and was associated with impaired angiogenic activities of diabetic ECFCs. Knockdown of miR-134–5p restored the impaired migration and angiogenic activities of diabetic ECFCs via upregulation of the nuclear receptor-interacting protein 1 (*NRIP1*). Furthermore, the study suggested that far-infrared radiation (FIR) pretreatment promoted the angiogenic ability of dysfunctional ECFCs induced by high glucose in a mouse ischemic limb model. The protective effects of FIR could be attenuated by the overexpression of miR-134–5p (93). The study indicated that miR-134–5p may serve as a novel target for improving dysfunctional diabetic ECFCs. However, the limited sample size in combination with the lack of long-term monitoring makes it difficult to confirm the therapeutic effect of FIR.

#### 4.2.2 Mesenchyme homeobox 2

Gohn et al. found that MEOX2 was upregulated in pregnancies complicated by T1DM and T2DM when compared with healthy pregnancies. *MEOX2* knockdown can reduce the tube formation of GDM-ECFCs. This study suggested that the upregulation of MEOX2 might be a compensatory mechanism to alleviate the impaired migration and angiogenesis in GDM-ECFCs (94). But the other researchers have revealed that high MEOX2 expression resulted in increased senescence (95) and less tube formation (96) in human umbilical vein endothelial cells (HUVECs). Thus, the protective role of *MEOX2* in GDM-ECFCs should be interpreted with caution.

#### 4.2.3 Transgelin

*TAGLN*, a TGF-β inducible gene expressed in smooth muscle cells, has been reported to be involved in the angiogenesis of endothelial cells (97). Varberg et al. evaluated the function of *TAGLN* in GDM-ECFCs and found that decreasing TAGLN expression contributed to improved migration and tube formation in GDM-ECFCs (11). These results suggest that knockdown of TAGLN could be a novel strategy to improve the function of GDM-ECFCs. Interestingly, they developed a bioactive nanoparticle that can conjugate to the surface of ECFCs. The nanoparticles, loaded with SB-431542 (a TGF-β inhibitor), can stably normalize TAGLN expression in GDM-ECFCs. Not only did the bioactive nanoparticles improve migration of GDM-ECFCs, but also they augmented the vasculogenesis of GDM-ECFCs *in vitro* and *in vivo* (98).

#### 4.2.4 Protocadherin 10

PCDH10, a member of the non-clustered protocadherins, is involved with adherens junctions (99). *PCDH10* has been found to be upregulated in GDM-ECFCs compared to ECFCs from normal pregnancies. The upregulation of *PCDH10* was due to the hypomethylation of the *PCDH10* promoter. Knockdown of *PCDH10* was able to rescue the defective proliferation, migration, cell adhesion, and angiogenic functions in GDM-ECFCs (12). Nevertheless, *PCDH10* was identified as a tumor suppressor gene in various cancers (99). Thus, it is necessary to assess the safety of knockdown of *PCDH10* in GDM-ECFCs.

### 4.3 Mesenchymal stem cells

In addition to specific biomolecules and genetic modification, co-injection with MSCs is an efficient strategy to boost the regenerative potential of ECFCs. MSCs are multipotent non-hematopoietic, fibroblast-like plastic adherent cells, which have the potential to differentiate into multiple cell types, such as chondrocytes, adipocytes, and osteocytes (100). MSCs have captured attention in vascular regeneration because these cells secrete large amounts of angiogenic factors, growth factors and cytokines (101). Several studies have demonstrated that combined transplantation of MSCs with healthy ECFCs exhibited stronger ability of vessel-like structures formation in mice when compared to translation of healthy ECFCs alone. The better effects of combined transplantation were due to the ability of MSCs to reduce the immune cell infiltration or their ability to produce paracrine factors (102, 103). However, there are very limited published data on the combination of ECFCs with MSCs in the context of DM. Lee et al. compared the vasculogenic capacity between ECFCs and ECFCs + MSCs under high glucose conditions (30 mM) in a diabetic immunodeficient mouse model. The results showed that ECFCs + MSCs potentiated the tube formation ability under hyperglycemic conditions compared to ECFCs alone. More importantly, the combination of ECFCs with MSCs was beneficial for forming tube functional microvessels *in vitro* (104). There are some advantages of combined transplantation of MSCs with ECFCs: (i) MSCs secrete a plethora of pro-angiogenic factors, such as VEGF-A and FGF2 (101); (ii) the immunomodulatory effects of MSCs protect against ECFCs rejection after transplantation (103); (iii) MSCs induce mesenchymal transition of ECFCs thorough NOTCH signaling, which improves engraftment and vasculogenic potential (105); (iv) MSCs bolster vasculogenic activity of ECFCs through the endoglin-mediated adhesion between MSCs and ECFCs (106).

### 4.4 Potential challenges and limitations of the above bioengineering strategies

Although the above strategies have shown promising results *in vitro* or in preclinical animal models, there are still some potential limitations that need to be considered. For pretreatment with biological compounds, this strategy is reliant on endogenous ECFC biology and its efficacy could be limited in defective ECFCs. More importantly, transient pretreatment may make it difficult to maintain the therapeutic effect for a much longer period. The use of genetically modified ECFCs will pose a biosafety concern. Viral transduction (adenovirus and lentivirus) is the most commonly used method for gene delivery into ECFCs because of its high and consistent transduction efficiency (107). Viral transduction with adenovirus and lentivirus has been associated with adverse effects such as toxicities, increased immunogenicity, and oncogenicity (108). As for co-injection with MSCs, it holds great potential for clinical translation because ECFCs and MSCs contribute to angiogenesis through different, yet complementary, mechanisms. However, the safety profile of combined therapy needs further research, as standardized approaches for combined therapy are not yet developed, including the optimal dosage, time, transplant type (autologous or allogenic), and route of administration.

## 5 Barriers to clinical translation of ECFCs and potential solutions

There is now significant evidence in pre-clinical models that ECFCs can be used as a therapy for diabetic vascular complications and that therapeutic effectiveness can be enhanced with modifications described above. However, it should be noted that there are no human clinical trials using ECFCs. The major challenges for clinical applications of ECFCs are divided into six different categories, including cell identity, cell dose, good manufacturing practice (GMP)-compliant cell manufacture, heterogeneity, efficacy, safety and cost (**Table 2**). First, it is necessary to establish unified surface markers for identifying ECFCs (37). Unified surface markers can improve the purity of ECFC populations, which may contribute to the production of a homogeneous cell population and facilitate superior comparison between studies using ECFCs. Single cell sequencing may further elucidate the characteristics of ECFCs and help to establish uniform surface markers. Uniform markers will also facilitate inter-study comparison and rapidly progress the ECFC field by reducing confusion and increasing knowledge. Second, the frequency of CB-ECFCs and PB-ECFCs is low (33, 34). ECFC large scale expansion strategies, e.g. bioreactor expansion, or enhancing ECFC potency is needed to achieve the desired clinical outcomes (109). Third, there is limited data in the literature describing GMP grade manufacture of ECFCs, with ECFC isolation and expansion being heavily reliant on high FBS content and the use of rat tail collagen for cell attachment (109). Collaboration between academic researchers, clinicians, and biotech companies can accelerate the development of GMP-compliant ECFCs. Fourth, ECFCs are an inherently heterogeneous cell population. The heterogeneity of ECFCs is affected by multiple factors, including the different donors (110), tissue origin (32, 111), and methods for isolation and culture of ECFCs (112). Therefore, it is difficult to reproducibly generate functionally equivalent ECFC populations. Fifth, disease status (113–115), cryopreservation (116), hypoxia and inflammatory microenvironment of transplantation sites (76) can lead to the loss of ECFC potency, which may impair therapeutic efficacy. The strategies mentioned in the previous paragraph should be explored to boost the potency of defective ECFCs. Lastly, the reagents (type I rat tail collagen and fetal bovine serum) for ECFC isolation and culture are not appropriate for clinical applications because they may carry animal-originated pathogens. The development of xeno-free medium might help to solve this issue, such as platelet lysate supplemented culture medium (109) and EC-Cult-XF ECFC medium (Stemcell Technologies, Canada). However, these xeno-free media are expensive to scale-up for clinical use currently. Collectively, there needs to be considerable attention paid to the good manufacturing practice (GMP) of human ECFCs keeping in mind the issues raised in this section on standardization, heterogeneity, optimal approaches for cell propagation, delivery and safety.

**Table 2:** Major Challenges for clinical applications of ECFCs.

Challenges	References
1. Identity: (a) Lack of unified surface markers for identifying ECFCs	(37)
2. Cell doses: (a) Low frequency of ECFCs; (b) Large-scale expansion methods need to be developed	(33, 34)
3. GMP-compliant cell manufacture:	(109)
4. Heterogeneity of ECFCs: (a) Different donors; (b) Different tissue origin; (c) Methods for isolation and culture of ECFCs	(32, 110–112)
5. Efficacy: (a) Defective function under disease status; (b) Loss of potency after cryopreservation; (c) Hypoxia and inflammation microenvironment of transplantation sites	(87, 113–116)
6. Safety and cost (a) Type I rat tail collagen or fetal bovine serum are derived from animal sources; (b) Toxicities, immunogenicity and oncogenicity induced by genetic engineering approaches (c) Allogeneic cell sources causing immunogenic responses (d) Xeno-free medium are expensive to scale-up for clinical use	(108, 109)

## 6 Comparison of ECFCs with other possible cell or EV treatments in diabetic vascular complication

Due to the low frequency of PB-ECFCs and impaired function of ECFCs in DM, other possible sources have been explored for the treatment of diabetic vascular complication. Thus, we next compared the efficacy of ECFCs with other stem cell approaches or extracellular vesicles (EVs) derived from stem cells in diabetic vascular complications (**Table 3**).

**Table 3:** Comparison of ECFCs with other possible cell or EV treatments in diabetic vascular complications.

Sources	Efficacy	Advantages	Limitations
ECFCs	Yes	Self-renewal ability and the capacity to form functional blood vessels	Low frequency; Defective function in diabetic autologous sources Immunogenic issues with allogeneic sources
ESCs	Unknown	Supreme proliferative capacity and pluripotent	Ethical controversies, allogeneic immune responses, and risk of teratoma formation
MSCs	Yes	Easy isolation and expansion; Reduces immune cell infiltration and produces immunomodulatory and angiogenic factors; Well tolerated and safe for clinical use	MSCs cannot directly form blood vessels; The immune suppression by MSCs may lead to the growth and spread of tumor cells
iPSCs-ECFCs	Unknown	Can be expanded to large numbers and low immunogenicity	The production is complex, time consuming, and costly; Risk of teratoma formation
MSC-EVs	Yes	Easy storage; Lower immunogenicity and an improved safety profile; Anti- inflammation and repair function in pre-clinical models	Lack of standardized isolation, characterization methods; Challenges of large-scale production
ECFC-EVs	Unknown	Easy storage; Lower immunogenicity and an improved safety profile	Lack of standardized isolation, characterization methods; Challenges of large-scale production

Pluripotent embryonic stem cells (ESCs) originate from the inner cell mass of the blastocyst, which possess the capacity to differentiate into any cell type (117). Animal studies have suggested that ESCs-derived endothelial cells can incorporate into the vasculature of the ischemic limb and improve limb perfusion (118, 119). These studies indicated that ESCs can successfully treat ischemia, while evidence of their effects in diabetic vascular complications is still lacking. A significant potential advantage of differentiating ESCs into endothelial cells lies in the opportunity to induce tissue-specific EC phenotypes (120). However, although ESCs exhibit a greater proliferative capacity than adult stem cells, the application of ESCs in clinical settings is hindered by ethical controversies over human embryos, allogeneic immune responses post transplantation, and the risk of teratoma formation (121).

MSCs from different sources have been widely used in the treatment of diabetic vascular complications. Preclinical models and clinical trials have demonstrated a broad range of beneficial effects of MSCs in the treatment for diabetic complications, including vascular protective effects (122–124). For instance, Zhang et al. summarized the effects of umbilical cord mesenchymal stem cells (UC-MSCs) in the treatment of diabetic foot, including nine preclinical experiments and five clinical trials. These studies have demonstrated the impressive efficacy and safety of UC-MSCs in diabetic foot (125). Besides, researchers have suggested that UC-MSCs and adipose-derived MSCs could have superior clinical application prospects as they are more easily accessible and have better immunomodulatory properties than MSCs from bone marrow (126, 127). Nonetheless, MSCs promote vascular repair through paracrine and trophic mechanisms instead of forming blood vessels themselves (128). The combination between MSCs and ECFCs could be an exciting therapeutic prospect.

Induced pluripotent stem cells (iPSCs) are derived from mature cells like fibroblasts, which can be reprogrammed to differentiate into any of the three germ layers (129). One recent breakthrough was the observation that human induced pluripotent stem cells (iPSCs) can be differentiated into ECFCs. These iPSCs-ECFCs have been shown to possess high clonal proliferative potential, form capillary structures in Matrigel, and contribute to vascular repair in both oxygen-induced retinopathy and hind limb ischemia mice model. The iPSCs-ECFCs have similar capacity of promoting neovascularization when they were compared with CB-ECFCs (130). More importantly, iPSCs-ECFCs were derived from fibroblasts, which were easily obtained in large quantities and did not face the immunologic barriers associated with allogeneic cell therapies. However, differentiation of iPSCs into the ECFC lineage is complex, time consuming, and costly. Undifferentiated cells during the differentiation process could cause teratomas and the potential of iPSCs-ECFCs needs to be further investigated in the context of DM.

EVs have attracted considerable attention due to the therapeutic potential of bioactive molecules contained inside EVs, including peptides, proteins, lipids, and nucleic acids (microRNAs and mRNA) (131). An additional factor to consider is that EVs have low immunogenicity and an improved safety profile compared to cell-based therapies (132). EVs from MSC are considered as promising products for the treatment of diabetic vascular complications [see review (133)]. In particular, MSC-EVs have been reported to promote angiogenesis and wound healing in diabetic mice model (134). MSC-EVs exert their protective effects in diabetic vascular complications through multiple methods, such as reduction of inflammatory cytokines, activation of angiogenesis, and promotion of collagen synthesis (135). Despite promising *in vitro* and *in vivo* results, to date no clinical trials have been conducted to evaluate the efficacy and safety of MSC-EVs in diabetic vascular complications. As for ECFC-EVs, a few studies suggested that they had protective effects on preclinical mouse models of ischemia, such as ischemic retinopathy (136) and ischemic kidney injury (137). However, no studies have yet been performed to investigate the therapeutic effects of ECFC-EVs in ischemic conditions in the context of a diabetic environment. Moreover, it remains unknown whether tissue source, isolation protocol, and disease status influence the bioactivity of ECFC-EVs.

Therefore, MSCs are the most studied cell therapy in diabetic vascular complications ECFCs have shown promising outcomes in preclinical studies. The combination MSCs with ECFCs, iPSCs-ECFCs and EVs from MSC and ECFCs offer promising avenues for treating diabetic vascular complications.

## 7 Conclusion and future perspective

ECFCs enhance the formation of blood vessels and contribute to revascularization, which may benefit diabetic patients with severe vascular complications. However, ECFCs isolated from DM patients exhibited a dysfunctional phenotype. Although the involvement of hyperglycemia, oxidative stress, and inflammation has been discussed in this review, underlying the mechanisms of dysfunctional ECFCs in DM are not well understood. The role of noncoding RNAs and epigenetic regulation in DM-ECFCs requires further study.

Currently, combining ECFCs with biological compounds, gene modification, or MSC has shown efficacy in rescuing diabetes-mediated ECFC damage. However these strategies need to be evaluated in more studies and pre-clinical models in the future. Besides, regulatory issues related to cell modification and issues of ECFC expansion under diabetic conditions will need to be addressed to enhance the realization of the potential of this therapy. Moreover, the application of biomaterials, 3D bioprinting of vascular structures might represent potential future strategies to improve the properties and functional capabilities of ECFCs in DM. The development of ipSCs-ECFCs and ECFC-EVs could be promising alternative treatments for patients with DM.

## Author contributions

YL: Conceptualization, Data curation, Funding acquisition, Investigation, Writing – original draft. CL: Methodology, Validation, Writing – review & editing. CA: Methodology, Validation, Writing – review & editing. TO: Conceptualization, Funding acquisition, Writing – review & editing.
